# Prolonged skin allograft survival by rM180 amelogenin in a murine skin transplantation model

**DOI:** 10.3389/fimmu.2025.1663437

**Published:** 2025-10-27

**Authors:** Miyu Shida, Terukazu Sanui, Karen Yotsumoto, Jinfeng Li, Mwannes Ahmad, Meng Xiao, Ziyu Wang, Chikako Hayashi, Yuki Nishimura, Takanori Shinjo, Takaharu Taketomi, Takao Fukuda, Fusanori Nishimura

**Affiliations:** ^1^ Department of Periodontology, Division of Oral Rehabilitation, Faculty of Dental Science, Kyushu University, Fukuoka, Japan; ^2^ Dental and Oral Medical Center, Kurume University School of Medicine, Kurume, Fukuoka, Japan

**Keywords:** amelogenin, allogeneic skin transplantation, Th1 cells, Th17/Treg balance, RNA sequencing analysis, M2 macrophages, immunosuppression

## Abstract

**Introduction:**

Amelogenin, used as a periodontal tissue regeneration material, promotes healing after periodontal surgery. A previous study has demonstrated that amelogenin is taken up by macrophages into the nucleus and inhibits major histocompatibility class II (MHC II) expression at the transcriptional level, thereby suppressing subsequent T cell activation. Therefore, in this study, we focused on the suppressive effect of amelogenin on MHC II expression and examined the effect of amelogenin on graft rejection following allogeneic skin transplantation in mice with different MHC II haplotype antigens.

**Methods and results:**

Skin grafts were treated with recombinant murine amelogenin (rM180) and transplanted into recipient mice. The rM180-treated group showed a significant increase in graft survival for up to 5.5 days and a lower necrotic score than the control group. Inflammatory cell infiltration and MHC II^+^ cells were significantly lower in the rM180 group. Furthermore, serum interferon-γ, interleukin-2, and interleukin-17A levels, splenic T-helper type 1 cells and helper type 17/regulatory T cells balance were reduced in the rM180 group. RNA sequencing analysis suggested "negative regulation of immune response" and "regeneration of myocytes and myofibrils" by amelogenin treatment. Among the upregulated genes in the rM180 group, *“POU domain class 2 transcription factor 2,” “lipocalin 2,”* and *“chitinase-like 4”* were ranked high. Additionally, the ratio of M2 macrophages significantly increased in rM180-treated grafts.

**Discussion:**

These results may suggest that amelogenin can be a safe immunosuppressant or therapeutic agent against autoimmune diseases without inducing unfavorable side effects.

## Introduction

The incidence of burns caused by accidents, fire-related incidents, and other factors has increased worldwide. According to the World Health Organization, approximately 11 million burns occur annually, resulting in approximately 180,000 deaths (1). Skin allografts are performed in patients who have lost a large amount of skin due to extensive burns or other diseases and who have no undamaged skin that can be used for autografting (2). Allografts are used to cover large areas of the body that have lost skin to reduce fluid and protein loss and prevent infection. Unlike other solid organ transplants, skin allografts are ultimately rejected; however, this allows for the formation of vascular-rich granulation on the skinless body surface, which facilitates the attachment of an autograft from the patient’s healed site. The acute rejection of allogeneic grafts that occurs during this process is primarily an attack that causes damage to the graft by an immunological response to foreign antigens on the graft, with major histocompatibility complex (MHC) antigens acting as the most important allogeneic antigens (3, 4). Several attempts have been made to extend the longevity of allografts and enhance their successful transition to autografts by using immunosuppressive agents. However, the use of immunosuppressants markedly increases the infection rate, and if discontinued, allografts are ultimately rejected (5, 6). Moreover, the long-term use of immunosuppressants poses a high risk of side effects, such as toxicity to the liver and kidneys or cancer, and severely limits the long-term survival of the patient (7). Therefore, novel local immunosuppressive drug delivery systems that allow long-term transplant survival without the need for systemic immunosuppressant administration are urgently needed.

Amelogenin belongs to the extracellular matrix family and is secreted by ameloblasts during tooth growth to promote hydroxyapatite crystal growth and enamel calcification. Additionally, amelogenin is involved in the development of periodontal tissues, including the cementum, through its deposition in the dentin of the tooth root. Based on the concept of mimicking tooth development, enamel matrix derivative (EMD) was developed. Amelogenin accounts for more than 90% of EMD and has been successfully used as a periodontal tissue regeneration material to regenerate the alveolar bone, which is lost as a result of periodontitis (8–10). Moreover, it is empirically known that the use of EMD in periodontal surgical procedures has a healing-promoting effect, reducing pain and swelling with a minimal inflammatory reaction after surgery (11). Additionally, it has been reported that amelogenin, the main component, exhibits anti-inflammatory effects (12). In our previous study, we performed a microarray analysis to compare unstimulated macrophages with those stimulated with rM180, a recombinant murine amelogenin, and reported that rM180 stimulation suppressed the gene expression of MHC class II (MHC II), which is important for antigen presentation in macrophages (13). We further demonstrated that rM180 translocates early into the nucleus of macrophages and suppresses the transcriptional activity of MHC II transactivator (CIITA), a transcriptional activator of MHC II molecules, resulting in a reduction in the synthesis and cell surface expression of MHC II, which in turn suppresses T-lymphocyte activation and reduces inflammation (14).

Based on these findings, the present study focused on the suppressive effect of amelogenin on MHC II expression and examined the effect of amelogenin on graft rejection by performing allogeneic skin transplantation between mice with different MHC II haplotype antigens.

## Materials and methods

### Animals

Male C57BL/6J (H-2D^b^) and BALB/c (H-2D^d^) mice, 6–8 weeks of age and weighing 20–25 g, were purchased from The Jackson Laboratory (Clea Japan, Tokyo, Japan). The mice were kept for at least one week on a 12-h light and dark cycle. All experiments were approved by the Animal Care and Use Committee of Kyushu University (Permit Number: A24-033-0) and were conducted in strict compliance with ethical guidelines.

### Preparation of recombinant murine M180 amelogenin

The cloning and expression of a glutathione S-transferase full-length M180 amelogenin fusion construct and the purification of rM180 have been described previously (14). The full-length mouse amelogenin (M180) cDNA was inserted into a vector and transformed into competent Escherichia coli. Bacterial pellets containing recombinant glutathione S-transferase-rM180 were harvested, and the fusion protein was cleaved on a column using PreScission protease (GE Healthcare, Boston, MA, USA) to obtain purified rM180. The removal of endotoxins from rM180 was verified (endotoxin level: < 0.03 EU per 10 µg of rM180).

### Skin transplantation

A 1.0 × 1.0 cm^2^ piece of skin from the back of a C57BL/6J mouse was transplanted onto the back of a BALB/c mouse. The recipient BALB/c mice were randomly divided into two groups: rM180 and control mice. Transplanted skin was treated with either PBS or rM180. The purified rM180 was adjusted to a concentration of 10 μg/100 μL with PBS, and 100 μL of the solution was applied dropwise evenly to the surface of the graft to be attached to the recipient. The transplanted skin was sutured at four points around the periphery using ETHICON COATED VICRYL^®^ Plus Antibacterial (polyglactin 910) Suture (Ethicon, Somerville, NJ, USA) as soon as possible after administered. From day 7 onward, the allografts were evaluated daily for skin necrosis by at least two observers in a blinded manner. Allograft rejection (necrotic score 0) was defined as spontaneous graft detachment. The necrotic areas (black spots) were evaluated according to the previous study by Zhao et al. (15). Briefly, they were roughly estimated by visual inspection with ImageJ 1.53 (NIH) as a supplementary tool, and six different score levels were defined according to the percentage of the necrotic area of the graft.

### *In vivo* antibody administration

To investigate the role of Lcn2, the mice received subcutaneous injections of either anti-mouse lipocalin 2/NGAL monoclonal antibody ([MAB1857]; R&D Systems, Minneapolis, MN, USA) or rat IgG2A isotype control antibody ([MAB006]; R&D Systems). Antibodies were diluted in sterile PBS, and 2 μg in a total volume of 4 μL was administered at four points around the graft site every 24 h for seven consecutive days, starting 24 h after transplantation.

### Histological analysis

Paraffin sections (thickness, 10 µm) of the skin graft tissues were deparaffinized using xylene and dehydrated with ethanol. Sections were stained with H&E or immunohistochemistry. Non-specific staining was blocked by incubation with Blocking One Histo (Nacalai Tesque, Kyoto, Japan) for 30 min at room temperature. These slides were incubated with the primary antibody, anti-rabbit CD4 antibody (ab287724; Abcam, Cambridge, UK), at 1:20 dilution, Anti-mouse CD8 alpha antibody (sc-7970, Santa Cruz Biotechnology, Dallas, TX, USA) at 1:250 dilution, Anti-MHC class II ([MRC OX-6]; Abcam) at 1:1000 dilution, CD19 Monoclonal antibody ([6OMP31]; Invitrogen, Carlsbad, CA, USA) at 1:500 dilution, F4/80 Monoclonal antibody ([BM8]; Thermo Fisher Scientific™,Waltham, MA, USA) at 1:500 dilution, Normal rat IgG (sc-2026, Santa Cruz Biotechnology) at 1:500 dilution, Mouse (G3A1) mAb IgG1 isotype control (5415S, Cell Signaling Technology) at 1:500 dilution, Rabbit (DA1E) mAb IgG XP isotype control (3900S, Cell Signaling Technology) at 1:500 dilution, Anti-Ym-1 + Ym-2 (Chil4) antibody ([EPR15263]; Abcam) at 1:200 dilution, Proteintech NGAL (Lcn2) polyclonal antibody (Proteintech Group Inc, Wuhan, China) at 1:400 dilution, Anti-OCT2 (Pou2f2) antibody (Sigma-Aldrich, St. Louis, CA, USA) at 1:500 dilution, or purified anti-Arginase1 antibody ([O94E6]; Biolegend, San Diego, CA, USA) at 1:200 dilution overnight at 4 °C in the dark. They were washed and incubated with a secondary antibody, and the nucleus was stained using SlowFade™ Diamond Antifade Mountant with DAPI (Thermo Fischer Scientific™). Photographs were taken using a BZ8000 (Keyence Co., Osaka, Japan), and the numbers of CD4, CD8, CD19, F4/80, MHCII, Chil4, Lcn2, and Pou2f2-positive cells were counted using a hybrid cell count application with BZ-X Analyzer software (Keyence Co). Images were analyzed using ZEISS LSM700 (Carl Zeiss, Oberkochen, Germany) and ZEN 2012 software. H&E staining was performed on allografts to assess tissue morphology.

### Tissue processing

Blood was collected from the facial vein using an Animal Lancet 5 mm (AS ONE, #21328703) and centrifuged at 2000 × *g* for 10 min to aspirate the serum. The spleens were passed through 70 μm cell strainers and centrifuged. The spleens were subjected to a round of red blood cell lysis. RBC Lysis Buffer (Biolegend, #420302) was used to lyse the erythrocytes. Skin grafts were harvested on day 4 or 6 post-transplant and processed into single-cell suspensions using Dri Tumor & Tissue Dissociation Reagent (BD Horizon™, # 661563) (BD Biosciences, San Diego, CA, USA).

### Serum cytokine measurement

Serum cytokine levels in peripheral blood were measured using the BD™ Cytometric Bead Array (CBA) Mouse Th1/Th2/Th17 CBA Kit purchased from BD Pharmingen™ (BD Biosciences).

### Flow cytometry

Freshly isolated spleen cells were obtained by gently milling the mouse spleens in PBS. Single-cell suspensions were washed with PBS and stained with live/dead fixable viability stain (Thermo Fisher Scientific™, # L34961). Fc receptors were blocked using Fc block (Biolegend, #156604) before surface staining with antibodies of interest (**Supplementary Table 1**) in FACS wash buffer (Biolegend, #420201; 10 min, 4 °C). The cells were washed and fixed with 1% formaldehyde. For intracellular cytokine or transcription factor assessment, cells were fixed and permeabilized with Cyto-Fast™ Fix/Perm Buffer Set (Biolegend, #426803) or Biolegend True-Nuclear™ Transcription Factor Buffer Set (Biolegend, #424401) before staining with antibodies targeting markers of interest. Isotype controls were used to confirm antibody specificity. The cells were incubated in the dark for 30 min at 4 °C and analyzed using a BD FACSLyric flow cytometer (BD Biosciences). Data were processed using BD FACSuite™ software v1.6 (BD Biosciences).

### RNA-seq

#### Sample preparation and next-generation sequencing analysis

RNA concentration was measured using a Nanodrop spectrophotometer, and RNA integrity (RIN value) and DNA contamination were assessed using an Agilent Technologies 2200 TapeStation equipped with an RNA ScreenTape. Total RNA samples with a concentration of >50 ng/µL and a RIN value > 7.0 were used for subsequent analyses.

Total RNA was treated to remove ribosomal RNA (rRNA) using the MGIEasy rRNA Depletion Kit, which employs rRNA-specific oligonucleotides to deplete rRNA and purify mRNA. The resulting mRNA was used for library preparation.

Libraries were generated using the MGIEasy RNA Directional Library Prep Set, which preserves RNA directional information. This information is crucial for understanding the transcriptional orientation of genes. The prepared libraries were sequence using the DNBSEQ-G400RS platform with paired-end reads of 150 base pairs each.

### Data analysis

The initial quality assessment of the raw sequencing data was performed using FastQC (version 0.11.9) to evaluate the overall quality of the data. Low-quality bases and adapter sequences were trimmed using Trimmomatic (version 0.36) to ensure clean and high-quality reads. The cleaned reads were mapped to the reference genome (GRCm39) using HISAT2 (version 2.1.0), which is a highly efficient and fast alignment program. The mapping results were used for the subsequent quantification. Reads were quantified using RSEM (version 1.3.0), which provides accurate and reliable quantification of gene and isoform expression levels. Bowtie2 was used as part of the RSEM workflow for alignment. Differential expression analysis was conducted using the EdgeR program, with a significance threshold of *P*-value < 0.05, to identify DEGs. Gene sets were categorized based on GO terms and KEGG pathways. Enrichment analysis was performed using the enrichplot package (version 1.16.1), and the results were visualized using ggplot2 (version 3.3.6). GSEA was used to detect variations in signaling pathways between the high and low expression groups. Background gene sets were sourced from the Molecular Signatures Database (MsigDB) version 7.0. Gene set size setting: Analysis is performed with a minimum of 15 and a maximum of 500. Differential pathway expression analysis was conducted, and significantly enriched gene sets were identified based on consistency scores with an adjusted *P*-value < 0.05. The protein interaction network was constructed using the STRING database (https://string-db.org/) version 12.0 and visualized using Cytoscape (16). This network analysis provides insights into the functional interactions between proteins, enhancing our understanding of the molecular mechanisms involved.

### Statistical analysis

All data are expressed as mean ± SD. Differences between the two groups were analyzed using Student’s t-test. Kaplan-Meier analysis was used to assess the differences between allograft survival curves and to calculate *P*-values. Statistically significance was set at *P*-value < 0.05. All statistical analyses were performed using GraphPad Prism version 10.4.1.

## Results

### rM180 amelogenin prolongs skin allograft survival and reduces necrosis levels

**Figure 1:**
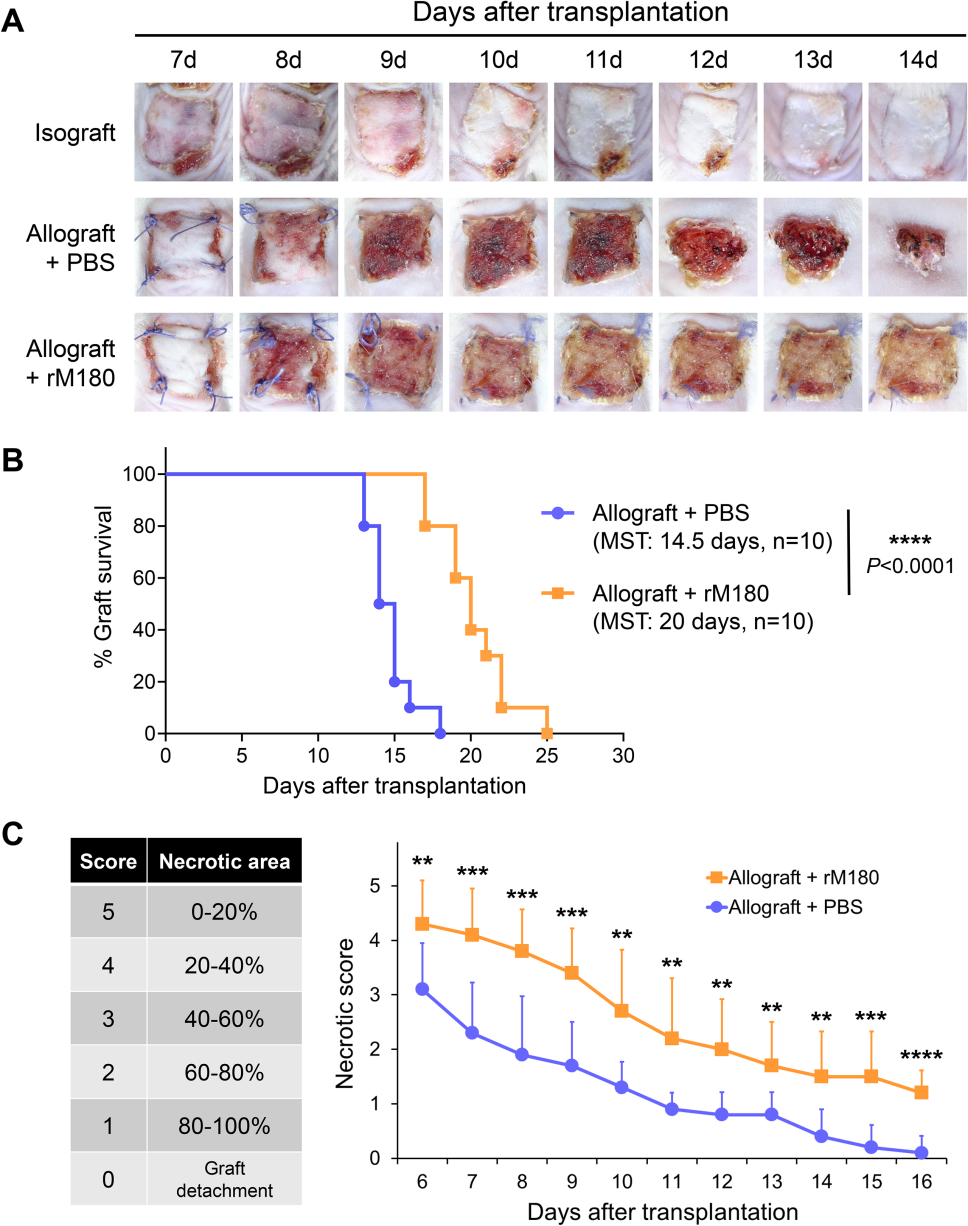
rM180 amelogenin prolongs the survival of skin allograft. **(A)** The photographs of the graft rejection have been shown in the isograft group (upper panel), PBS-treated allograft group (middle panel), and rM180-treated allograft group (lower panel) from day 7 to 14 post-transplantation. **(B)** Kaplan-Meier survival curves of allografts from rM180 treatment (n = 10) and PBS-treated (n = 10) groups by day of post-transplantation. The data were analyzed using GrafPad Prism 10.4.1. The significance of differences between groups was determined using log-rank tests; ****P < 0.0001. **(C)** Statistical analysis of necrotic levels of grafts from day 6 to 16 post-transplantation. Different score levels indicate different necrotic areas of skin allografts treated with rM180 or PBS. The significance of differences between groups was determined using a two-tailed unpaired Student’s test; **P < 0.01; ***P < 0.001; ****P < 0.0001. Data represent mean ± SD. Similar results were obtained in ten independent experiments. PBS, phosphate-buffered saline; MST, median survival time.

We established a mouse skin graft model to investigate the effects of amelogenin on graft rejection. A total 10 μg of recombinant mouse amelogenin (rM180) was applied to the dorsal skin (1.0 × 1.0 cm^2^) harvested from C57BL/6J donor mice and applied to the dorsal recipient bed of MHC-mismatched BALB/c recipient mice. The skin grafts were observed daily from days 7 to 14 post-transplantation and documented photographically. The syngeneic skin grafts satisfactorily adhered within two weeks (**Figure 1A**, upper panel), whereas most of the skin grafts in the control group, to which phosphate-buffered saline (PBS) was applied, were significantly reduced in size at approximately day 12 and were eventually rejected (**Figure 1A**, middle panel). Moreover, graft rejection in the rM180-applied group (rM180 group) was delayed by a median survival time of 5.5 days compared with that in the control group (**Figure 1A**, lower panel, and **Figure 1B**), and the necrotic area (black spots) of skin grafts in the rM180 group was consistently smaller than that in the control group from days 6 to 16 post-transplantation (**Figure 1C**).

### rM180 suppresses inflammatory cell infiltration into allogeneic skin grafts

**Figure 2:**
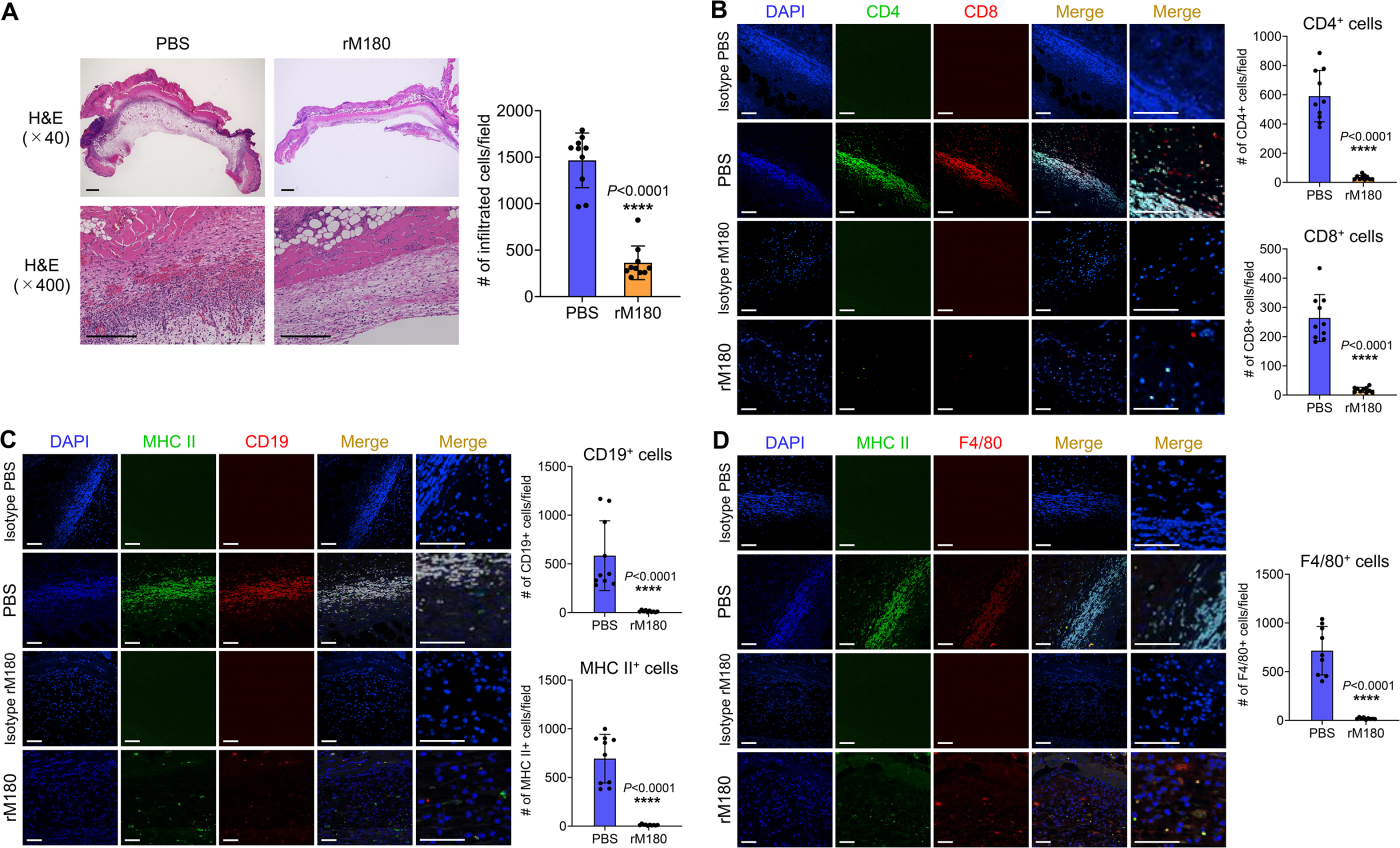
Transplantation with rM180 reduces T cell, B cell, and macrophage infiltration into the allograft skin. The skin allografts were harvested at seven days post-transplantation. **(A)** Representative images of H&E staining of allografts from the rM180 and PBS groups. Quantification of H&E staining corresponding to the two groups on day 7. Scale bars: 500 μm. **(B–D)** Representative images of immunohistofluorescence staining for CD4 and CD8 **(B)**, CD19 and MHC II **(C)**, F4/80 and MHC II **(D)**, or isotype controls in the rM180 and PBS groups. Quantification of immunohistofluorescence staining corresponding to the two groups on day 7. Scale bars: 500 μm. The significance of differences between groups was determined using a two-tailed unpaired Student’s test; ****P < 0.0001. Data represent mean ± SD. Similar results were obtained in ten independent experiments. H&E, hematoxylin and eosin.

To explore the effects of rM180 on the skin graft surroundings, inflammatory cell infiltration, and tissue damage were determined using histopathology of the skin grafts seven days after transplantation. The PBS-treated skin grafts showed histological signs of rejection and necrosis and were thick and swollen. Massive inflammatory cell infiltration was observed at the interface between donor and recipient skin. However, grafts in the rM180 group were thinner and had significantly fewer infiltrating cells (**Figure 2A**). The infiltration of T lymphocytes (CD4^+^, CD8^+^), B lymphocytes (CD19^+^), macrophages (F4/80^+^), and antigen-presenting cells (MHC II^+^) was determined by immunohistofluorescence analysis. In the grafts from the control group, clusters of CD4^+^ and CD8^+^ T cells (**Figure 2B**), CD19^+^ B cells (**Figure 2C**), F4/80^+^ macrophages (**Figure 2D**), and MHC II^+^ cells (**Figures 2C, D**) were detected seven days after transplantation. However, the frequencies of these clusters in grafts from the rM180 group were significantly lower (**Figures 2B–D**).

### rM180 reduces the serum interferon-gamma (IFN-γ), interleukin (IL-2), and IL-17 levels after allogeneic skin transplantation

**Figure 3:**
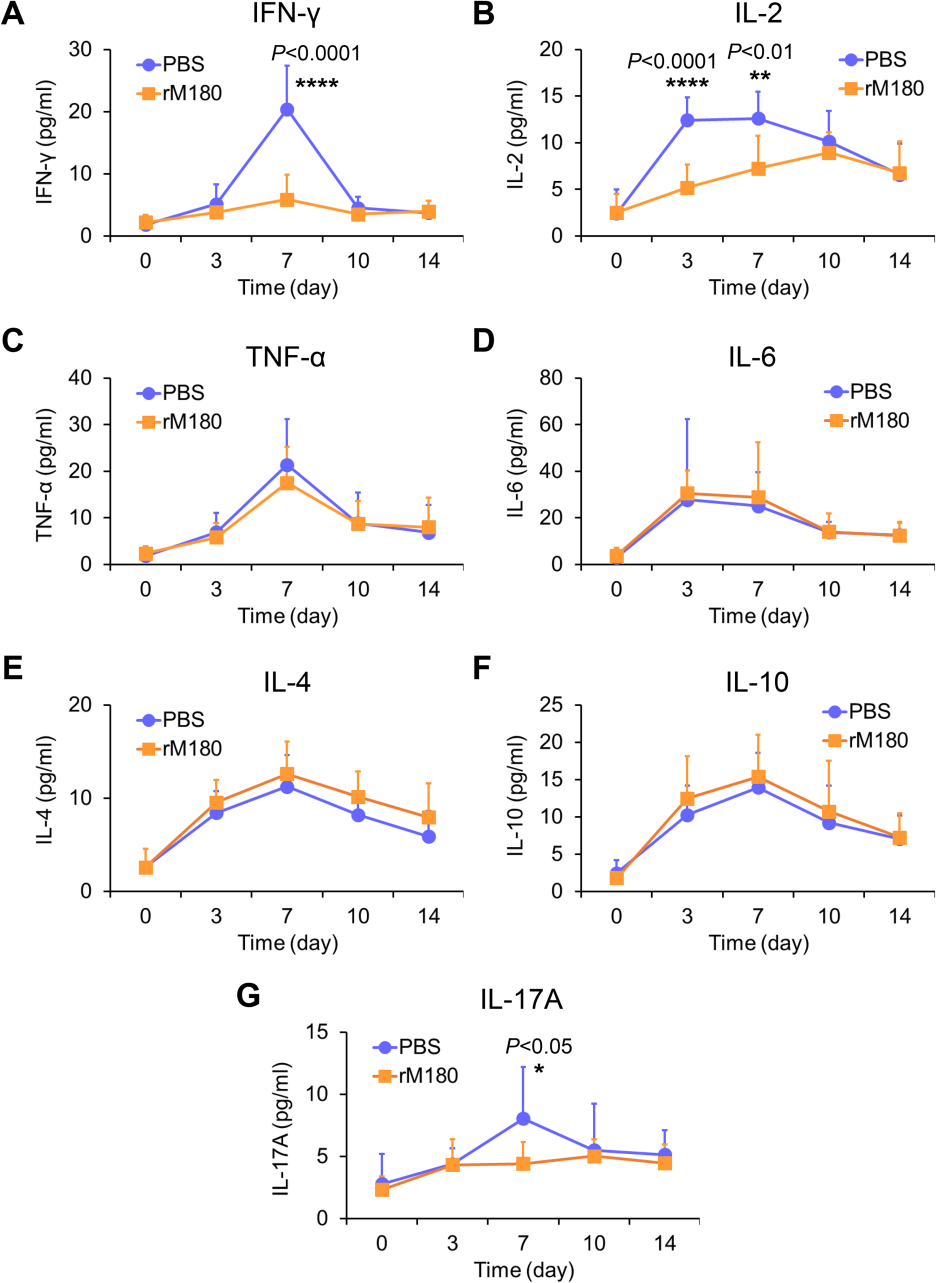
Determination of cytokine secretion in the serum of mice after skin transplantation with rM180. Peripheral blood was taken at the indicated times after allogeneic skin transplantation. The levels of IFN-γ **(A)**, IL-2 **(B)**, TNF-α **(C)**, IL-6 **(D)**, IL-4 **(E)**, IL-10 **(F)**, and IL-17A **(G)** were measured using flow cytometry. The significance of differences between groups was determined using a two-tailed unpaired Student’s test; *P < 0.05; **P < 0.01; ****P < 0.0001. Data represent mean ± SD. Similar results were obtained in ten independent experiments.

To study the effect of rM180’s potent inhibition of inflammatory cell infiltration in skin grafts on peripheral tissues, serum cytokine secretion levels were analyzed using flow cytometry. The concentration of cytokines peaked on day 7 post-transplantation, except for IL-2 and IL-6, which reached their peak on day 3 post-transplantation. In particular, the levels of the T-helper type 1 (Th1) cytokines IFN-γ (**Figure 3A**) and IL-2 (**Figure 3B**) in the serum of the rM180 group were much lower than those of the control group. On day 7 post-transplantation, the serum IFN-γ level in the rM180 group was only 30% of that in the control group, and on day 3 post-transplantation, the serum IL-2 level in the rM180 group was approximately 40% of that in the control group. This finding suggests the rM180-induced Th1 cell differentiation and dysfunction after allogeneic skin grafting. In contrast, no significant differences in the tumor necrosis factor-alpha (TNF-α) (**Figure 3C**) and IL-6 (**Figure 3D**) levels were observed between the two groups. In the rM180 group, the serum levels of IL-4 (**Figure 3E**) and IL-10 (**Figure 3F**) were slightly higher than those in the control group at all time points; however, no significant differences were observed. Furthermore, serum IL-17A levels in the rM180 group on day 7 after skin grafting were significantly lower than those in the control group (**Figure 3G**).

### Local administration of rM180 to allogeneic skin graft sites decreases inflammation in the spleen

**Figure 4:**
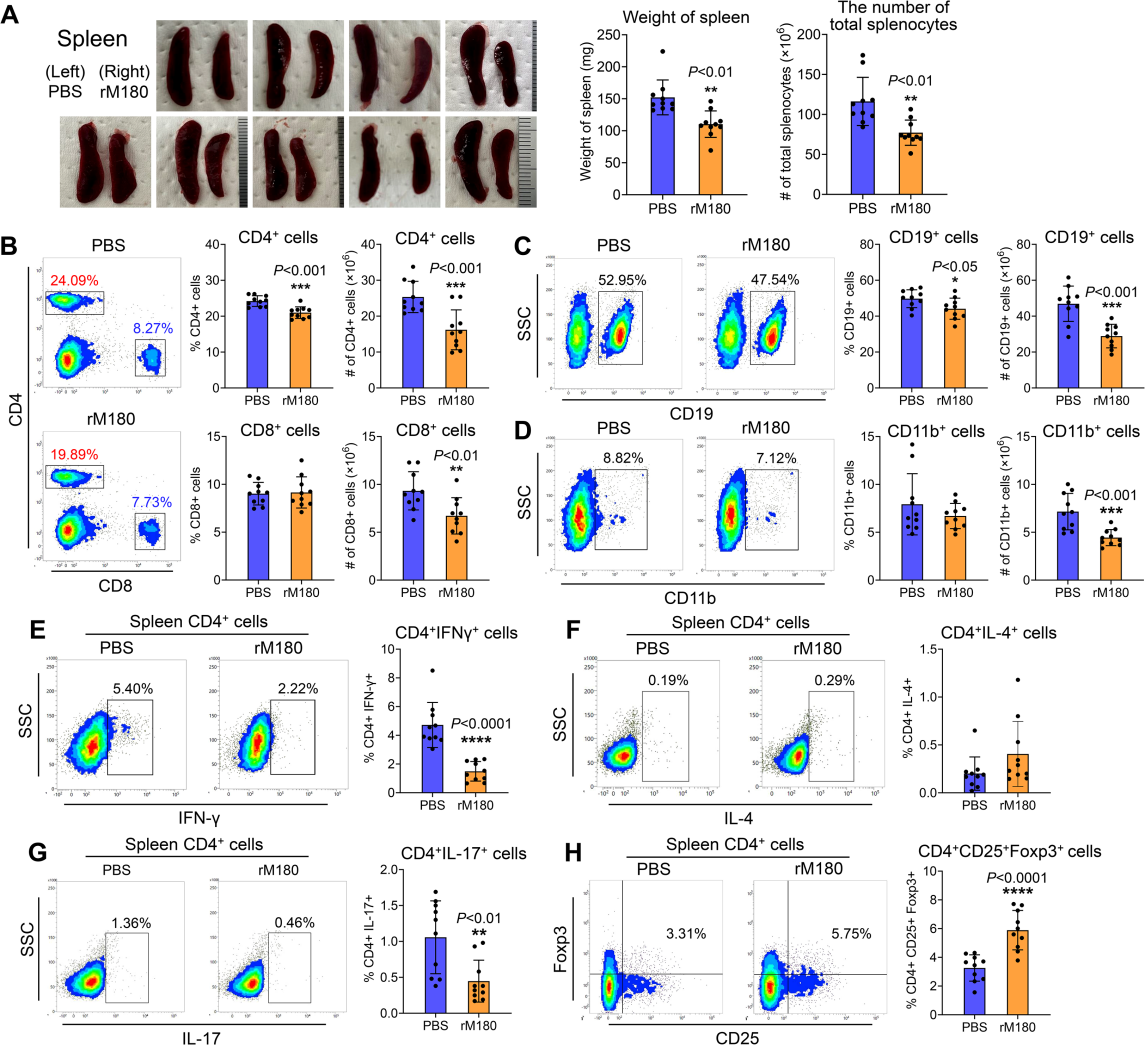
Local application of rM180 is associated with less severe inflammation in the spleen. **(A)** The photographs of the spleens have been shown in the rM180 group (right) and the control group (left) from day 7 post-transplantation. Quantification of the weight of the spleen and the number of total splenocytes corresponding to the two groups on day 7. **(B–D)** Flow cytometry was used for quantification of the percentages and the number of CD4^+^ and CD8^+^ T cells **(B)**, CD19^+^ B cells **(C)**, and CD11b^+^ macrophages **(D)** in the spleen of the rM180 and control groups. **(E–H)** All plots were gated on live CD4^+^ T cells. Representative plots and bar graphs display the percentages of IFN-γ^+^ **(E)**, IL-4^+^ **(F)**, IL-17^+^ **(G)**, and CD25^+^ Foxp3^+^ **(H)** cells in the spleen of the rM180 and control groups. The significance of differences between groups was determined using a two-tailed unpaired Student’s test; *P < 0.05; **P < 0.01; ***P < 0.001; ****P < 0.0001. Data represent mean ± SD. Similar results were obtained in ten independent experiments.

Because local administration of rM180 suppressed Th1 and Th17 cytokine levels in the peripheral blood, we investigated its effect on the spleen, a secondary lymphoid organ, after allogeneic skin transplantation. As shown in **Figure 4A**, splenic hypertrophy was observed in the control group on day 7 post-transplantation, whereas the spleens of the rM180 group exhibited a significant decrease in total weight and cell count. The spleens of the rM180 group were slightly larger than those in the wild type mice without skin transplantation (**Supplementary Figure 1**). Flow cytometric analysis revealed lower percentages of CD4^+^ and CD19^+^ cells in the spleens of the rM180 group than in those of the control group (**Figures 4B, C**). Conversely, the two groups did not exhibit significant differences in the percentages of CD8^+^ and CD11b^+^ cells (**Figures 4B, D**). However, the cell count of each cell population in the spleen was significantly lower in the rM180 group than in the control group (**Figures 4B–D**). As shown in **Figure 3**, IFN-γ, IL-2, and IL-17A concentrations in the peripheral blood of the rM180 group on day 7 post-transplantation were reduced. Therefore, we examined CD4^+^ cell subsets population in the spleen. The results revealed that the percentage of IFN-γ-positive cells in splenic CD4^+^ cells in the rM180 group was approximately 30% of that in the control group at 7 days post-transplantation (**Figure 4E**) and that the IL-17-positivity rate decreased to almost 50% (**Figure 4G**). In contrast, there were no differences in the percentage of IL-4 positivity in splenic CD4^+^ cells between the two groups (**Figure 4F**), and the percentage of splenic CD4^+^Foxp3^+^CD25^+^ cells in the rM180 group was almost twice that of splenic CD4^+^Foxp3^+^CD25^+^ cells in the control group (**Figure 4H**). These data suggest that local administration of rM180 resulted in decreased differentiation of CD4^+^ cells into Th1 and Th17 cells in the peripheral blood and increased differentiation into regulatory T (Treg) cells. This may contribute to the immune tolerance and delayed graft rejection by rM180 observed after skin transplantation.

### Effect of rM180 on the transcriptional profiles involved in skin grafting

**Figure 5:**
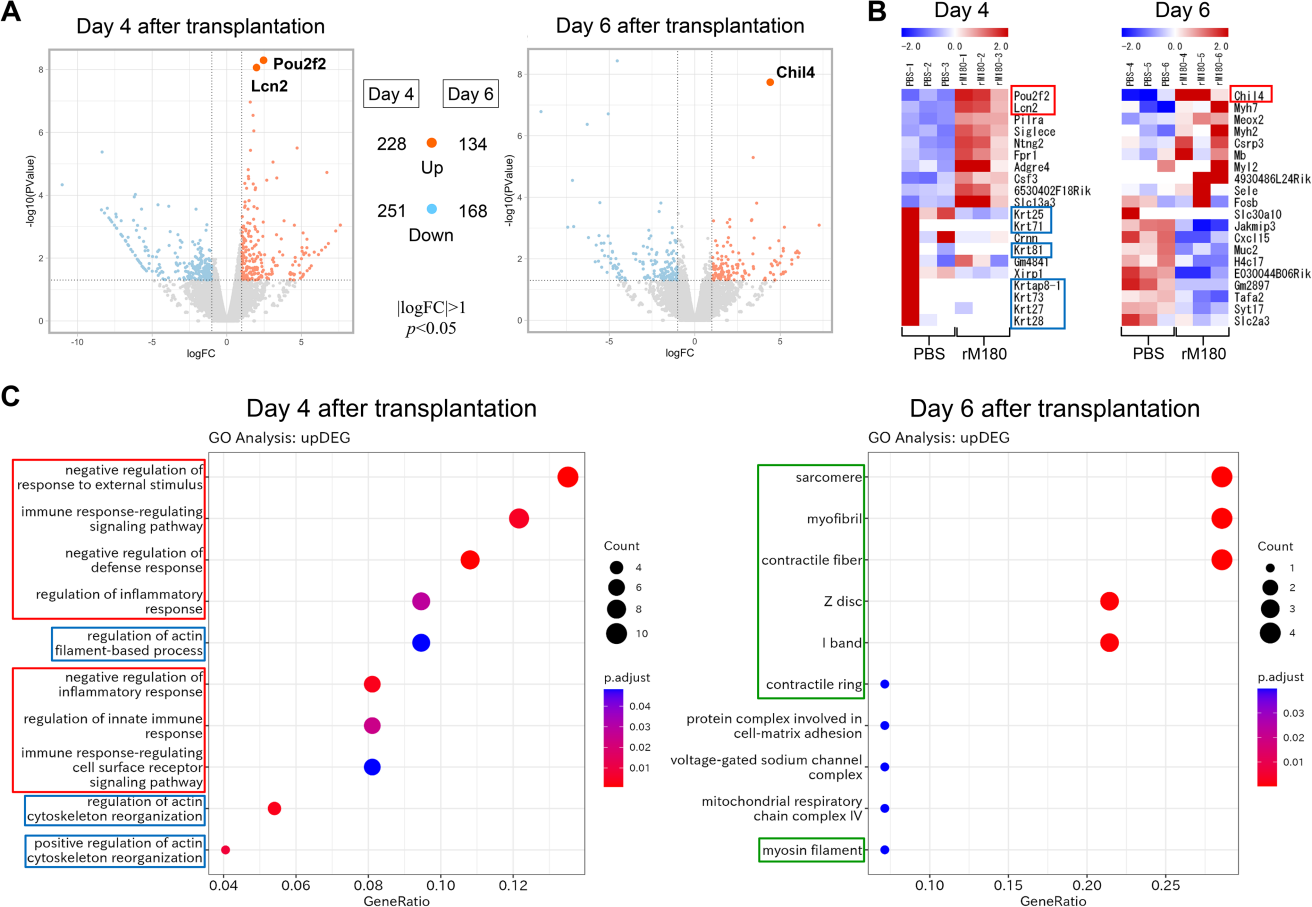
Impacts of rM180 treatment on the transcription profile on skin graft. **(A)** DEG volcano distribution map. The orange color represents upregulated transcripts. The light blue represents downregulated transcripts. (|logFC|>1, *P*< 0.05) **(B)** Hierarchical clustering heat map of DEGs (n = 3) in each group. **(C)** GO and KEGG analysis of the role of upregulated DEGs and screening enrichment pathway on day 4 or day 6 post-transplantation. Select the 10 most significant KEGG pathways to draw a scatter diagram for display. The abscissa is a ratio of number of differential genes annotated to the KEGG pathway to the total number of differential genes, the ordinate is the description of the KEGG pathway, the size of the dot represents the number of genes annotated to the KEGG pathway, and the color from red to blue represents enrichment of the saliency size.

As shown in **Figure 3**, rM180 potently suppressed IL-2 production in peripheral blood from day 3 post-transplantation, while its systemic anti-inflammatory effects had largely disappeared after day 10. We therefore hypothesized that rM180 exerts an anti-inflammatory effect on the progression of inflammatory response that occurs immediately after transplantation. To investigate the potential molecular mechanisms of the rM180-induced prolongation of skin graft survival, RNA sequencing (RNA-seq) analysis of skin grafts from the rM180 group was performed on day 4 and 6 post-transplantation. Compared with the control group, 479 differentially expressed genes (DEGs), including 228 upregulated and 251 downregulated genes, were detected in the rM180 group on day 4 post-transplantation, and 302 DEGs, including 134 upregulated and 168 downregulated genes, were detected in the rM180 group on day 6 post-transplantation (**Figure 5A**). POU domain class 2 transcription factor 2 (*Pou2f2*) and lipocalin 2 (*Lcn2*) were among the genes that exhibited the most upregulated expression on day 4 post-transplantation, and chitinase-like protein 4 (*Chil4*) exhibited the most upregulated expression on day 6 post-transplantation (red box) (**Figures 5A, B**). In contrast, the expression of keratin-related genes (blue box) was highly downregulated on day 6 post-transplantation (**Figure 5B**). Inflammation and immune responses associated with skin graft rejection resulted in increased keratinization, suggesting that rM180 suppresses rejection-induced keratinization.

Gene Ontology (GO) and Kyoto Encyclopedia of Genes and Genomes (KEGG) pathway analyses were performed to further investigate the functions of the upregulated DEGs and the pathways involved in graft protection via rM180. The top 10 molecular functions are shown in **Figure 5C**. GO and KEGG analyses demonstrated that DEGs in the rM180-treated group were associated with “negative regulation of immune response” (red box) and “actin cytoskeleton reorganization” (blue box) on day 4 post-transplantation. On day 6, the genes that exhibited a change in expression levels in the rM180-applied group were mainly associated with “myocytes and myofibrils” (framed in green) (**Figure 5C**). Based on the above-mentioned analysis, the rM180-mediated prolongation of graft survival in skin graft models may be associated with the modulation of the inflammatory response within skin grafts and the regeneration of muscle fibers. The results of the KEGG analysis of inversely downregulated DEGs are shown in **Supplementary Figure 2**.

To investigate the anti-inflammatory mechanism of rM180 in prolonging the survival of murine skin grafts, gene set enrichment analysis (GSEA) was performed. We demonstrated that T and B cell receptor signaling pathway, phagocytosis, natural killer cell-mediated cytotoxicity, toll-like receptor signaling pathway, Janus kinase/signal transducer and activator of transcription pathway and chemokine signaling pathway, apoptosis, and allograft rejection were downregulated on day 4 post-transplantation (**Supplementary Figure 3A**), whereas hedgehog signaling pathway and transforming growth factor β were upregulated at a higher level in the rM180 group than in the control group (**Supplementary Figure 3B**). Thus, GSEA on day 4 post-transplantation suggested that most pathways involved in rM180-mediated prolongation of skin graft survival were related to the regulation of the immune–inflammatory response. In contrast, the expression of genes associated with cardiomyopathy and myocarditis was downregulated on day 6 post-transplantation (**Supplementary Figure 3C**). This suggests that rM180 suppresses the destruction and degeneration of muscle fibers.

### Immunosuppressive effect of the rM180-induced enhanced expression levels of *Pou2f2* and *Lcn2* at day 4 post-transplantation

**Figure 6:**
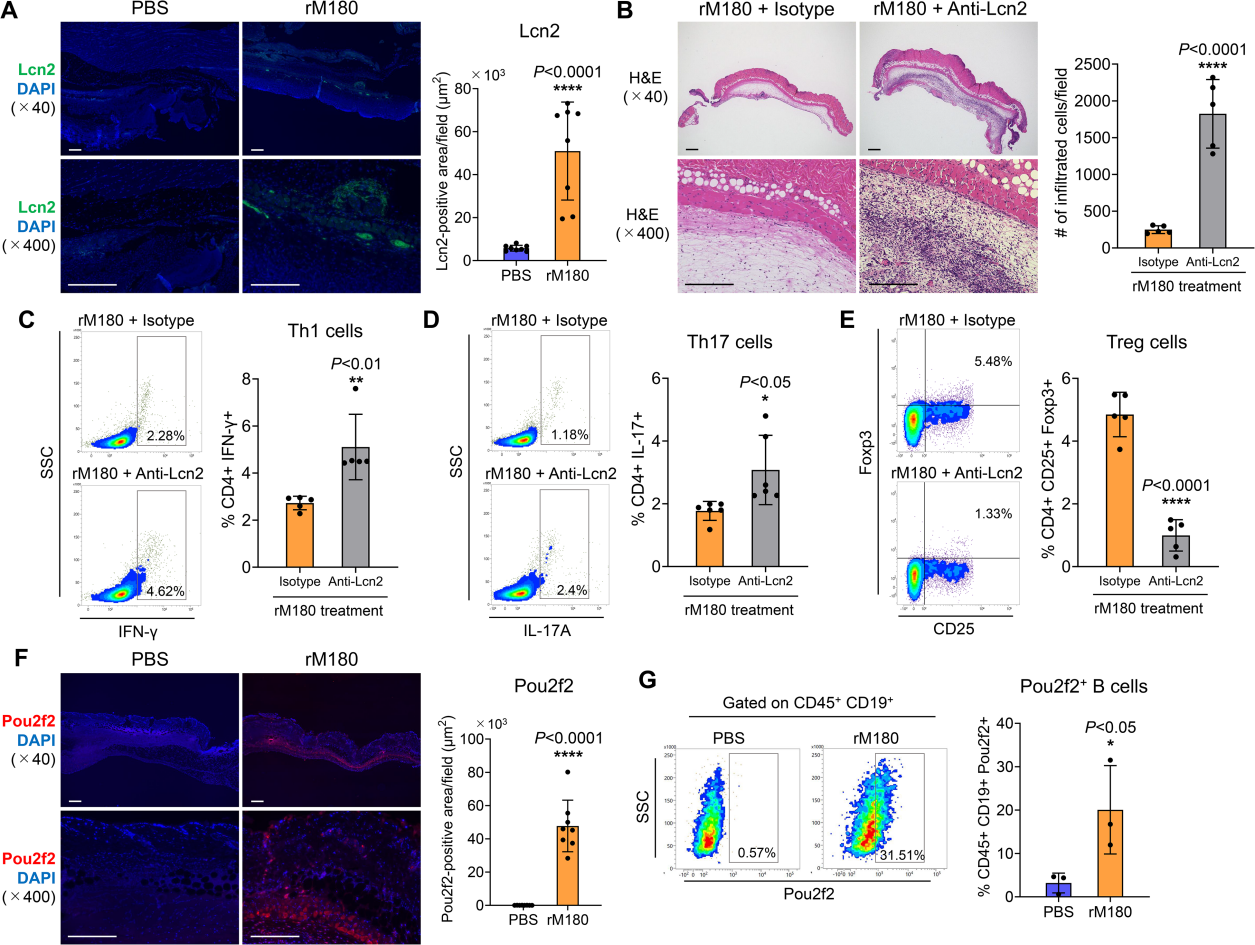
Lcn2 and Pou2f2 expression in skin allografts. **(A)** The skin allografts were harvested at four days post-transplantation. Representative images immunohistofluorescence staining for Lcn2 of allografts in the rM180 and PBS groups. Quantification of immunohistofluorescence staining corresponding to the two groups on day 4. Scale bars: 500 μm. **(B)** Representative images H&E staining of allografts from the rM180 group injected with neutralizing antibody targeting Lcn2 (anti-Lcn2) or isotype control. Quantification of H&E staining corresponding to the two groups on day 7. Scale bars: 500 μm. **(C–E)** All plots were gated on live CD4^+^ T cells. Representative plots and bar graphs display the percentages of IFN-γ^+^ **(C)**, IL-17^+^ **(D)**, and CD25^+^ Foxp3^+^ **(E)** cells in the spleen of the rM180 group injected with anti-Lcn2 or isotype control on day 7. **(F)** Representative images of immunohistofluorescence staining for Pou2f2 of allografts in the rM180 and PBS groups. Quantification of immunohistofluorescence staining corresponding to the two groups on day 4. **(G)** All plots were gated on live CD45^+^ CD19^+^ cells using flow cytometry. Representative plots and bar graph display the percentages of Pou2f2^+^ B cells in skin allografts of the rM180 and control groups. The significance of differences between groups was determined using a two-tailed unpaired Student’s test; *P < 0.05; ****P < 0.0001. Data represent mean ± SD. Similar results were obtained in eight **(A, F)**, five **(B–E)**, or three **(G)** independent experiments.

RNA-seq analysis demonstrated that *Pou2f2* and *Lcn2* were the most upregulated genes in the rM180 group 4 days post-transplantation (**Figure 5B**). Lcn2 is an acute-phase protein secreted by immune and epithelial cells in mucosal tissues. It is a 25-kD protein that covalently binds to matrix metalloproteinase-9 and is expressed in cells such as macrophages. In response to inflammation induced by various stimuli, Lcn2 levels increase and have been reported to mediate both pro- and anti-inflammatory responses (17). One study also has reported that Lcn2 promotes the process of skin wound healing in response to growth factors (18). First, immunostaining of grafts with Lcn2 demonstrated strong expression of Lcn2 in blood vessels or lymphatic vessels at the border between the rM180-applied skin graft and the recipient area (**Figure 6A**). To investigate whether the anti-inflammatory effects of amelogenin were caused by the Lcn2, anti-Lcn2 antibody, a neutralizing antibody against Lcn2, was injected around the graft every other day 24 h after amelogenin application. When the group injected with anti-Lcn2 antibody after application of rM180 was compared with the group injected with isotype control, hematoxylin and eosin (H&E) staining analysis 7 days after skin grafting confirmed that the grafts in the anti-Lcn2 group were thicker and had infiltrated immune cells compared with the isotype control group (**Figure 6B**). The size of the spleen in the rM180 group was increased by the anti-Lcn2 neutralizing antibody (**Supplementary Figure 4**), and the decrease in the proportion of Th1 and Th17 cells in the spleen and the increase in Treg in the rM180 group was inhibited (**Figures 6C–E**). These results suggest that amelogenin-induced Lcn2 restricts the migration of inflammatory cells into the graft and reduces the proportion of Th1 and the Th17/Treg ratio in peripheral lymphoid tissues.

Pou2f2, also known as Oct2, is a B cell-regulatory transcription factor belonging to the POU domain family that uses the POU domain to bind to DNA (19, 20). Pou2f2 functions as a transcription factor that plays a pivotal role in B cell proliferation and differentiation by binding to the octamer DNA motifs present in the promoter of the immunoglobulin gene, and represses the expression of immunoglobulin in B cells (21). Next, we analyzed the distribution of Pou2f2 in skin grafts and observed a strong fluorescent signal of Pou2f2 in a band at the boundary between the graft and recipient bed in the rM180-applied group but not in the control group (**Figure 6F**). Furthermore, B cells in the skin grafts of the rM180 group strongly expressed Pou2f2 compared with those in the control group (**Figure 6G**).

### rM180 enhances Chil4 expression and induces M2 macrophage differentiation at the recipient site of the skin graft

**Figure 7:**
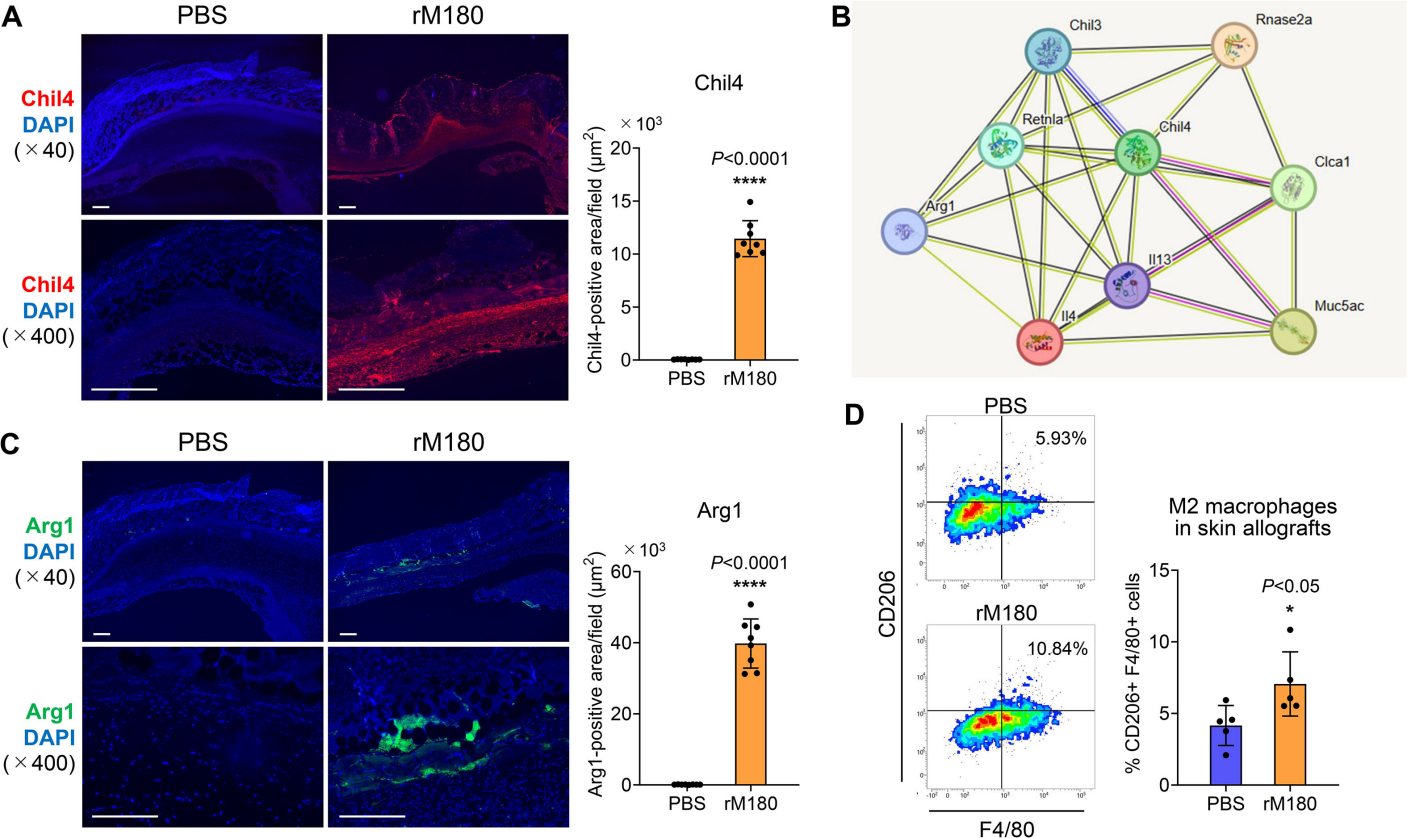
rM180 enhanced Chil4 expression and induced M2 macrophage polarization in skin graft recipients. The skin allografts were harvested at six days post-transplantation. **(A, C)** Representative images immunohistofluorescence staining for Chil4 **(A)** and Arg1 **(C)** of allografts in the rM180 and PBS groups. Quantification of immunohistofluorescence staining corresponding to the two groups on day 6. Scale bars: 500 μm. **(B)** PPI analysis and screening of the hub gene and key signaling pathways in DEGs. **(D)** Representative plots and bar graphs display the percentages of CD206^+^F4/80^+^ M2 macrophages in skin allografts of the rM180 the control groups using flow cytometry. The significance of differences between groups was determined using a two-tailed unpaired Student’s test; *P < 0.05; ****P < 0.0001. Data represent mean ± SD. Similar results were obtained in eight **(A, C)** or five **(D)** independent experiments.

Based on the results of the RNA-seq analysis demonstrating that Chil4 expression was the strongest on day 6 post-transplantation, immunostaining with Chil4 was performed on the grafts. The results demonstrated a strong fluorescent staining band of Chil4 at the graft recipient site in the rM180 group but not in the control group (**Figure 7A**). To investigate which proteins Chil4 interacts with and are involved in biological processes and signaling pathways, we performed protein–protein interaction analysis based on the results of RNA-seq analysis and identified nine hub genes, including upregulated genes such as *Chil4*, *Chil3*, *resistin like alpha* (*Retnla*), *IL-4*, *IL-13*, *ribonuclease A family 2A* (*Rnase2a*), *chloride channel accessory 1* (*Clca1*), *Mucin 5 subtype AC* (*Mus5ac*), and *Arginase 1* (*Arg1*) (**Figure 7B**). Macrophages are broadly classified into inflammation-induced M1 and wound-healing M2 cells. M1 macrophages are activated by lipopolysaccharides and other factors and produce proinflammatory factors, such as inducible nitric oxide synthase, TNF-α, IL-1β, and IL-6 (22, 23). In contrast, M2 macrophages are induced by IL-4 and IL-13, express CD206 and Arg1, and produce anti-inflammatory cytokines such as transforming growth factor-β, which are responsible for angiogenesis, removal of apoptotic cells, resolution of inflammation, and tissue repair (24–27). Thus, the macrophage is involved in both destruction and regeneration and plays an important role in the interface between inflammation and tissue regeneration. The M2 macrophage-related hub genes identified here are thought to be primarily involved in the negative regulation of the immune response, as demonstrated by the KEGG analysis (**Figure 5C**). As shown in **Figure 7C**, there was a strong expression of Arg1, an M2 macrophage marker, interspersed between rM180-applied grafts and the recipient bed, compared with the control group. Furthermore, flow cytometric analysis revealed that M2 macrophages significantly increased in skin grafts treated with rM180 (**Figure 7D**). These results suggest that M2 macrophages induced by rM180 may be responsible for tissue repair and graft protection at 6 d post-transplantation.

## Discussion

**Figure 8:**
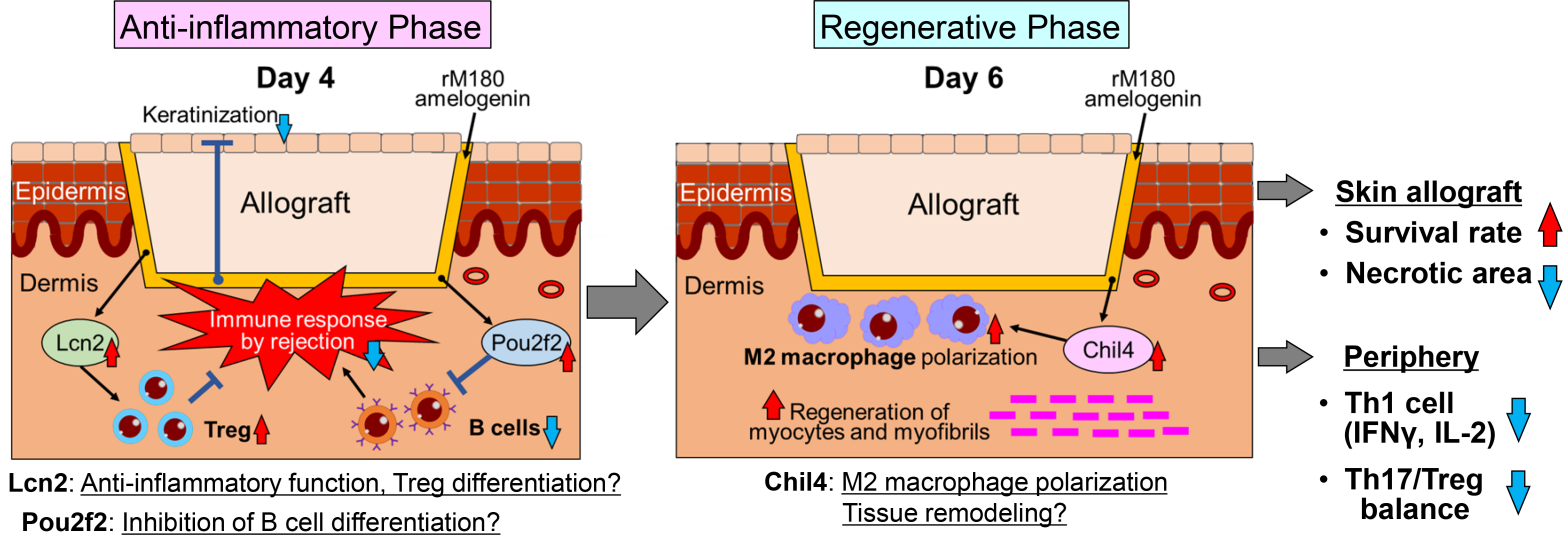
Proposed mechanisms of delayed rejection of skin allograft by rM180 amelogenin. The pretreatment of rM180 on mouse skin allografts may suppress rejection-induced hyperkeratosis and activate Lcn2 and Pou2f2 expression, which negatively regulates the immune response on day 4 post-transplantation. In particular, Lcn2 may play a role in inducing differentiation into Treg cells, while Pou2f2 may play a role in suppressing differentiation into B cells. On day 6 post-transplantation, rM180 enhances Chil4 expression and polarizes macrophages toward an M2 phenotype, suggesting that may repair muscular tissue. This causes a delayed rejection and a reduction in necrosis levels of skin allografts by rM180, thereby decreasing the percentage of CD4^+^ T cells, particularly, the percentage of Th1 cells and the Th17/Treg ratio in the periphery.

In the present study, pretreatment with rM180 suppressed skin graft rejection, resulting in reduced necrosis and prolonged survival of mouse skin allografts. Additionally, bioinformatic analysis revealed that on day 4 post-transplantation, the immune response was mainly suppressive, suppressing graft hyperkeratosis, which is a characteristic of rejection, and that Lcn2 and Pou2f2 further negatively regulated the immune response. In particular, Lcn2 may play a role in inducing Treg cell differentiation, whereas Pou2f2 may play a role in suppressing B cell differentiation. On day 6 post-transplantation, Chil4 expression was enhanced by the application of rM180 to the grafts, suggesting that macrophages differentiate into the M2 type and simultaneously regenerate myocytes and myofibrils. Consequently, the percentage of CD4^+^ T cells decreased in the periphery; that is, the percentage of Th1 cells decreased, and the Th17/Treg ratio decreased (**Figure 8**).

In other studies using animal skin grafts, new immunosuppressant candidates were administered by intraperitoneal, subcutaneous, or repeated intravenous injections. In contrast, in this study, a single application of rM180 to skin grafts prolonged rejection by 5.5 days. Additionally, there were no apparent systemic side effects of rM180 during this period. In clinical dentistry, EMDs containing 90% rM180 have been on the market for over 20 years, and no adverse reactions have been reported in over 2 million cases of periodontal tissue regeneration therapy in 44 countries. We have previously reported that rM180 migrates to the macrophage nucleus within 5 min and inhibits the transcriptional activity of CIITA by suppressing H3K27ac and H3K4me3 on histone H3 within the CIITA p-IV region, thereby selectively suppressing the cell surface expression of MHC II molecules, resulting in the attenuation of T-cell activity (14). Since the suppression of MHC II expression by rM180, in this case, is decreased by approximately 50%, this alone does not explain the strong suppression of immune cell infiltration in skin grafts by rM180 observed in this study, indicating the possible involvement of another immunosuppressive mechanism. T cells primarily drive allogeneic transplant rejection. Although all components of the innate and adaptive immune systems are involved in graft rejection, T lymphocytes, especially CD4^+^ T cells, are the most important in this process (28). Once activated, CD4^+^ T cells primarily recruit and activate other effector cells, such as macrophages, CD8^+^ T cells, and B cells (29, 30). Low serum levels of IL-2, IFN-γ, and IL-17A indicate partial defects in Th1 and Th17 cell differentiation and function. In acute rejection, Th1 cells predominantly infiltrate the graft and produce IL-2 and IFN-γ, and IFN-γ induces expression of MHC II molecules and activation of B cells (30, 31). In a model of acute rejection, IFN-γ-/- mice demonstrated delayed skin graft rejection (32). Although allograft rejection is traditionally associated with Th1 differentiation, recent studies have shown that Th17 cells and IL-17 are also closely associated with allograft rejection (33, 34).

It has been reported that Lcn2, which was strongly expressed in rM180-treated grafts on day 4 post-transplantation, reacts with receptors on various cell types and exerts biological effects on cell migration, adhesion, and morphological changes in immunocompetent cells (35, 36). Furthermore, it has been suggested that Lcn2 plays a role in Treg cell proliferation (17, 37) and promote polarization toward M2 macrophages in an IL-10/signal transducer and activator transcription pathway 3-dependent manner (38). In a previous study, we have reported that the stimulation of macrophages with rM180 enhanced the expression of the M2 markers CD163 and CD206 in a time-dependent manner and promoted their differentiation into M2 macrophages accompanied by morphological changes to a spindle shape (39). We also demonstrated that rM180, an extracellular molecule, induces changes in the microenvironment of the cell adhesion surface of macrophages and induces their differentiation into M2 macrophages via cytoskeletal remodeling (39). These results were consistent with the aforementioned cellular functions of Lcn2. Additionally, Lcn2 promotes skin wound healing, but its efficacy is markedly reduced by local treatment with Lcn2-blocking antibodies (36), and *Lcn2*-knockout mice exhibited enhanced systemic and local inflammation and delayed skeletal muscle regeneration after femoral artery ligation (40). Lcn2 may be involved in the suppression of the immune response on day 4 post-transplantation and in the regeneration of myocytes and myofibrils on day 6 post-transplantation, as observed in this study. A series of studies have also demonstrated that Pou2f2 regulates B cell function and suppresses antibody production through the induction of miR-210 (41). The decreased percentage of splenic CD19^+^ cells among Pou2f2 cells observed in the rM180 group in this study may be attributed to this function.

Furthermore, on the fourth day after transplantation, the gene group related to “cytoskeleton remodeling” was increased by rM180. In our previous study, proteome analysis detected many cytoskeleton-related proteins, such as amelogenin-associated molecules (42), and further demonstrated that rM180 promotes the activation of Rac1, a small GTP-binding protein, by associating with Grp78, a heat shock protein, thereby promoting lamellipodia formation in periodontal ligament cells, providing a driving force for cell migration (43, 44). These results suggest that amelogenin directly binds to the cytoskeleton, partially activates remodeling partially via Lcn2, and controls various cell functions.

Chil4, also known as Ym2, was highly expressed in rM180-treated grafts on day 6 after transplantation and belongs to the chitinase-like protein family (45). Several studies have demonstrated the involvement of chitinase-like protein in tissue regeneration (46–48). Chil4 is primarily expressed in the stomach, followed by the lungs (49), particularly in the stratified squamous epithelium of the upper alimentary tract, in some chief and parietal cells of the glandular stomach, and in the olfactory and respiratory nasal epithelium (45), where it has been suggested to play an important role in hematopoiesis and tissue remodeling (50). Chil4 is also produced by macrophages and stimulated by Th2 cytokines such as IL-4 and IL-13 (51, 52) and has been reported as a potential protein biomarker for allergic asthma (53–56). Although the exact mechanism of Chil4 function remains unclear, it has been reported that injury to the adult olfactory epithelium induces upregulation of Chil4 in supporting cells and promotes regeneration of the olfactory epithelium (57), suggesting that tissue remodeling may be mediated by Chil4 in skin grafts six days after transplantation in this study.

This study has two major limitations. First, while RNA-seq analysis detected increased gene expression of Lcn2, Pou2f2, and Chil4 by rM180 and their protein expression was also confirmed, the direct correlation between these molecules and the immunosuppressive effects remains unexplored. Although a neutralizing antibody against Lcn2 partially demonstrated a causal link between rM180-mediated immunosuppression (suppressing peripheral Th1/Th17 differentiation while increasing Treg cells), this evidence is not sufficient. Furthermore, the potential effects on cytoskeletal remodeling and muscle tissue regeneration are purely speculative based on KEGG analysis. Future work should focus on targeting immune cells affected by amelogenin (e.g., Th cells, B cells, and macrophages) and include further molecular and cellular analyses including signal transduction experiments. Additionally, because amelogenin rapidly altered gene clusters in the skin graft within just two days, single-cell RNA-seq (scRNA-seq) on targeted immune cells, combined with trajectory or pseudo-time analysis, is necessary to fully elucidate the dynamics of amelogenin-mediated immune cells in the context of graft rejection and immunosuppression. Second, while we demonstrated that local rM180 application reduces systemic inflammation, the mechanism of this systemic effect is unclear. Given that amelogenin is a high-molecular-weight protein that forms particulate aggregates under physiological conditions (58, 59), it is unlikely to be absorbed directly into the bloodstream. A more probable mechanism is that amelogenin-stimulated immune cell populations in the graft circulate systemically and migrate to organs like the spleen. Thus, the systemic effect may be secondary to the reduction of local graft inflammation. Future studies should include measurements of rM180 concentration in blood and its tissue distribution to clarify this mechanism.

Overall, amelogenin may be a safe immunosuppressant with no obvious side effects and is a potential therapeutic agent for autoimmune diseases or allergies.

## Data Availability

The datasets presented in this study can be found in online repositories. The names of the repository/repositories and accession number(s) can be found in the article/**Supplementary Material**.
