# Supplementary figures and images for: Prolonged skin allograft survival by rM180 amelogenin in a murine skin transplantation model
Source: Front Immunol. 2025 Oct 27;16:1663437. doi: 10.3389/fimmu.2025.1663437 (PMC12597935; doi:10.3389/fimmu.2025.1663437)

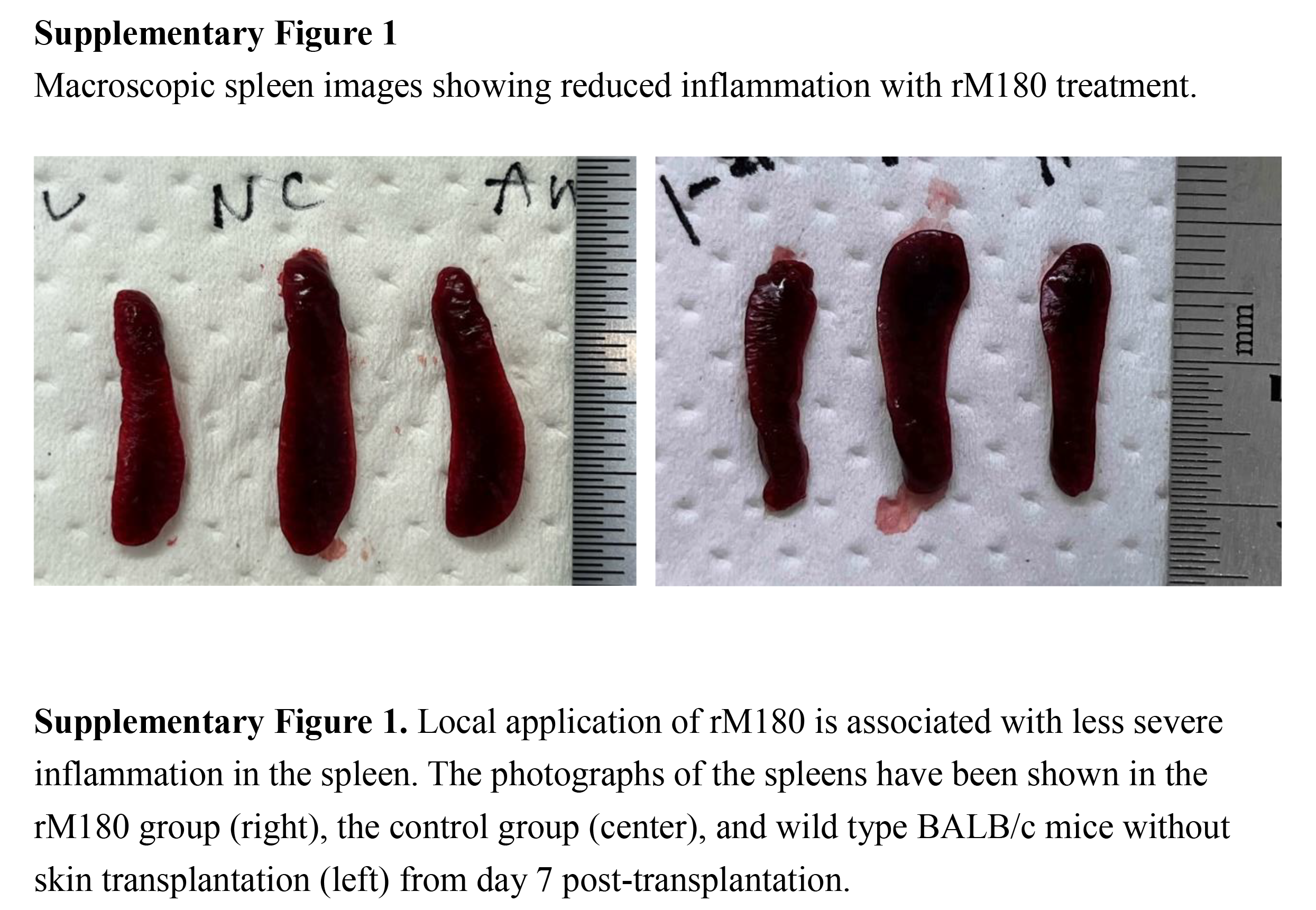

Supplement: Supplementary file 1 [file Image1.tif]

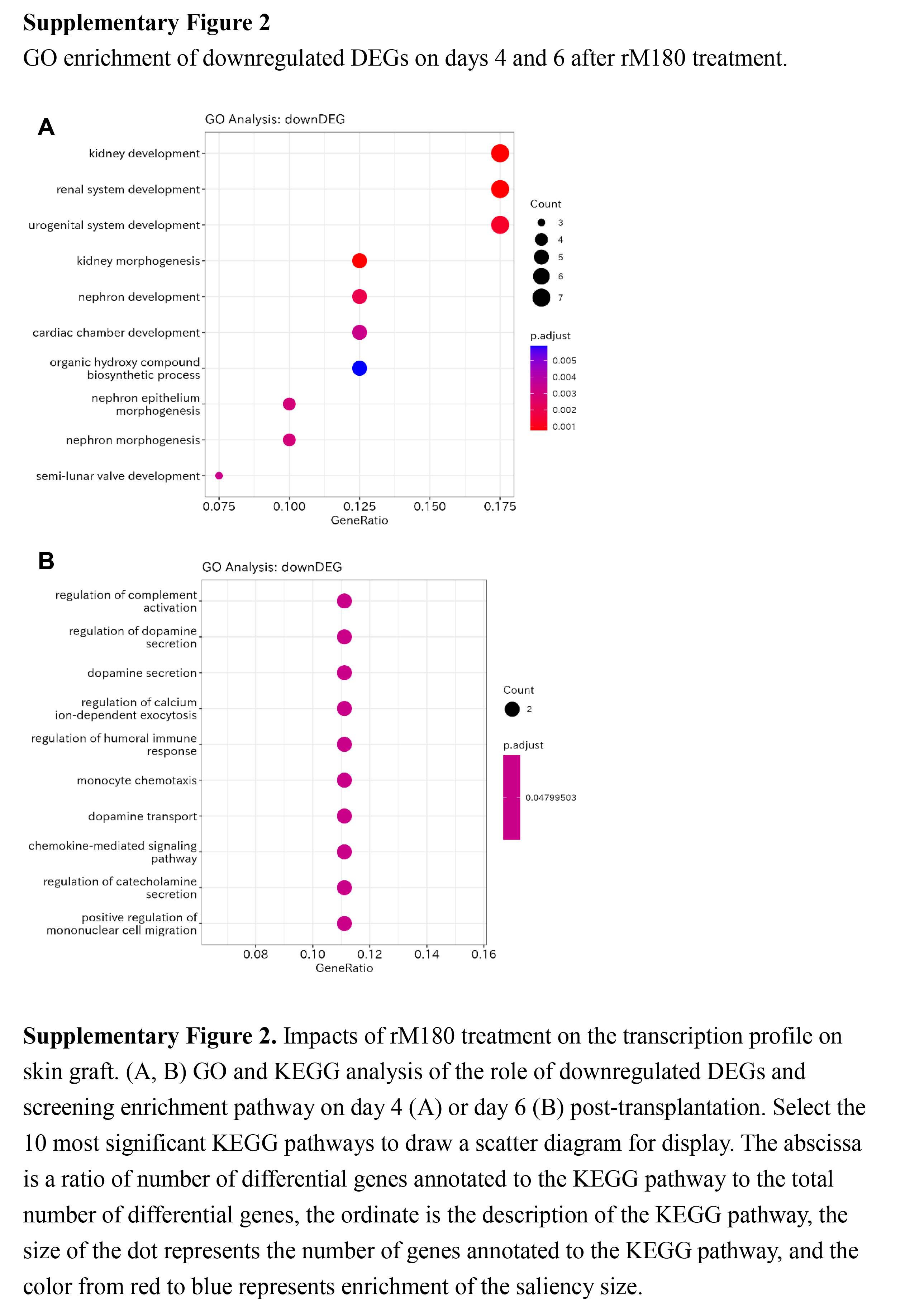

Supplement: Supplementary file 2 [file Image2.tif]

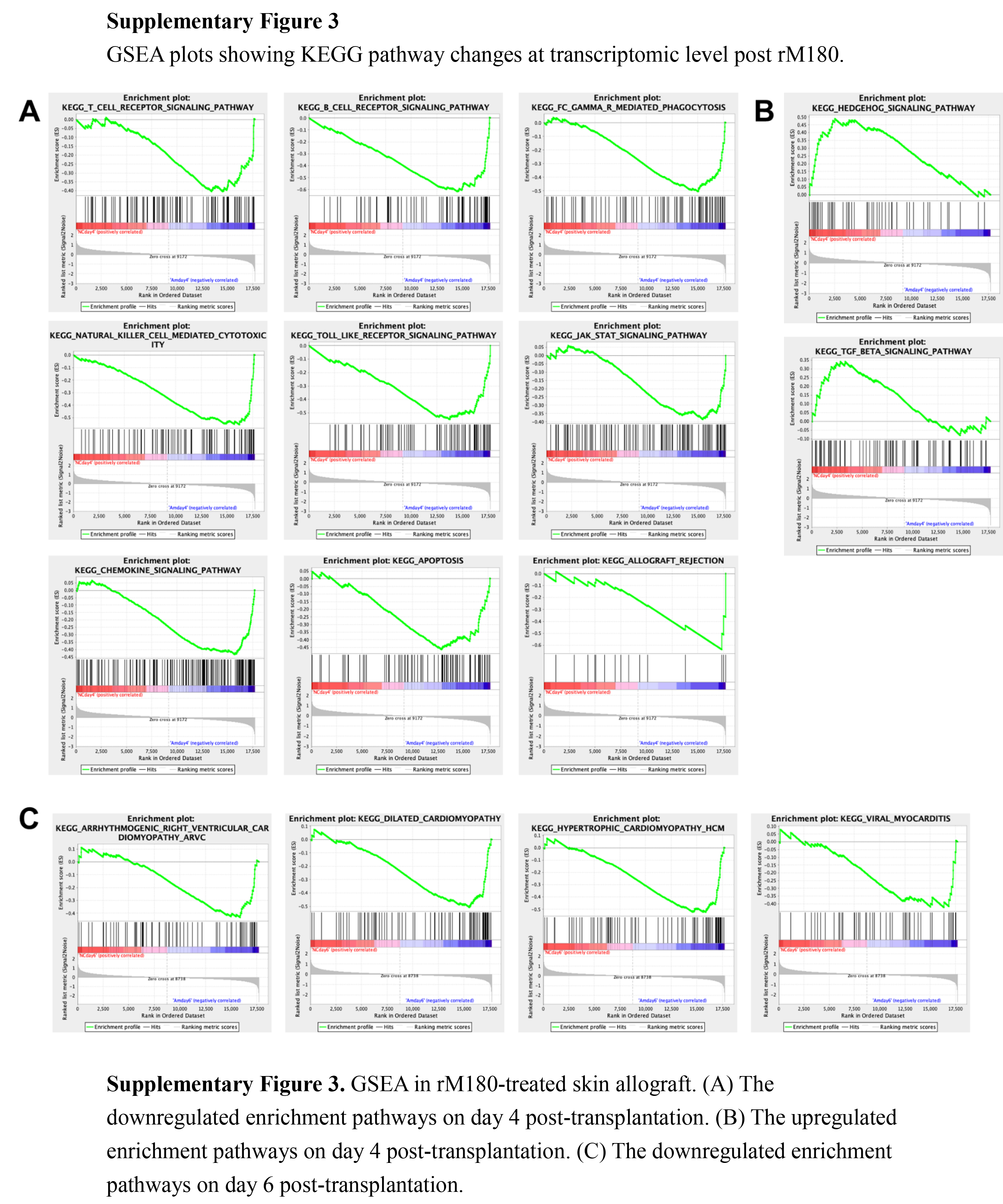

Supplement: Supplementary file 3 [file Image3.tif]

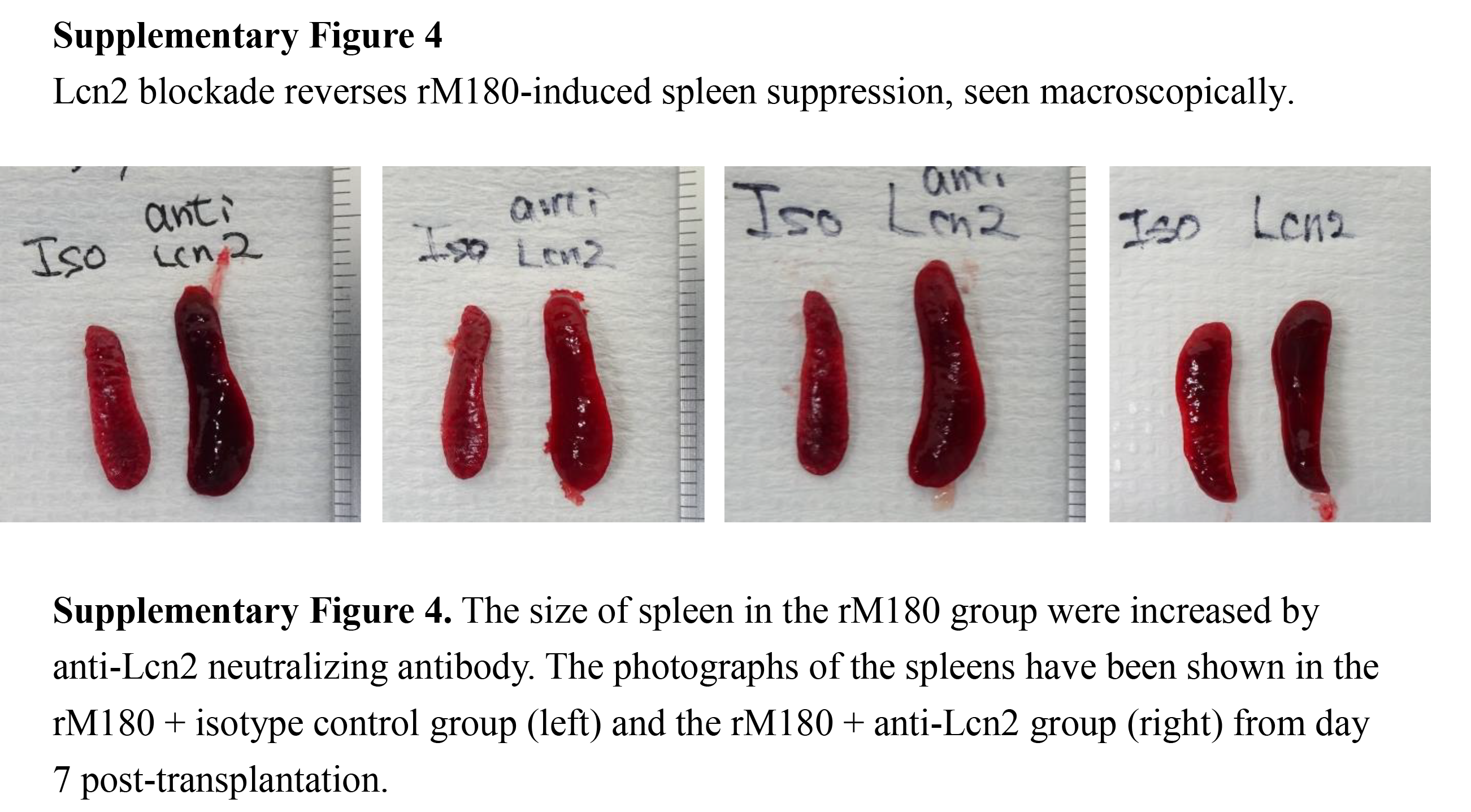

Supplement: Supplementary file 4 [file Image4.tif]
