# Supplementary material for: Prolonged skin allograft survival by rM180 amelogenin in a murine skin transplantation model
Source: Front Immunol. 2025 Oct 27;16:1663437. doi: 10.3389/fimmu.2025.1663437 (PMC12597935; doi:10.3389/fimmu.2025.1663437)
Supplement: Supplementary file 5 [file Table1.docx]

**Supplementary Table 1**

Comprehensive list of antibodies used for flow cytometric staining assays.

**Supplementary Table 1.** Antibodies used for flow cytometric analysis
